# Perceived Motion and Operational Momentum: How Speed, Distance, and Time Influence Two-Digit Arithmetic

**DOI:** 10.3389/fpsyg.2021.653423

**Published:** 2021-07-13

**Authors:** Maciej Haman, Hubert Młodzianowski, Michał Gołȩbiowski

**Affiliations:** ^1^Faculty of Psychology, University of Warsaw, Warsaw, Poland; ^2^University of Warsaw, Warsaw, Poland

**Keywords:** operational momentum, mental arithmetic, mental number-line, numerical cognition, spatial-numerical associations, representational momentum, spatial attention

## Abstract

Operational momentum was originally defined as a bias toward underestimating outcomes of subtraction and overestimating outcomes of addition. It was suggested that these estimation biases are due to leftward attentional shift along the mental number-line (spatially organized internal representation of number) in subtraction and rightward shift in addition. This assumes the use of “recycled” mechanisms of spatial attention, including “representational momentum” – a tendency to overestimate future position of a moving object, which compensates for the moving object’s shift during preparation of a reaction. We tested a strong version of this assumption directly, priming two-digit addition and subtraction problems with leftward and rightward motion of varied velocity, as velocity of the tracked object was found to be a factor in determining representational momentum effect size. Operands were subsequently moving across the computer screen, and the participants’ task was to validate an outcome proposed at the end of the event, which was either too low, correct, or too high. We found improved accuracy in detecting too-high outcomes of addition, as well as complex patterns of interactions involving arithmetic operation, outcome option, speed, and direction of motion, in the analysis of reaction times. These results significantly extend previous evidence for the involvement of spatial attention in mental arithmetic, showing movement of the external attention focus as a factor directing internal attention in processing numerical information. As a whole, however, the results are incompatible with expectations derived from the strong analogy between operational and representational momenta. We suggest that the full model may be more complex than simply “moving attention along the mental number-line” as a direct counterpart of attention directed at a moving object.

## Introduction

The term “operational momentum” refers to two biases (probably closely linked) found in mental arithmetic. First, people have a tendency toward underestimating the outcomes of subtraction and overestimating the outcomes of addition when exact counting is prohibited (we refer to this as “magnitude operational momentum”). Second, people also show a tendency toward associating subtraction with the left side of their attentional space and addition with the right side (“directional operational momentum”). These two biases were shown to coincide (Knops et al., 2009b) and have been typically regarded as two manifestations of the same process: moving internal attention along the “mental number line” (a spatially organized mental representation of number).

### Operational Momentum as a Spatial-Directional Bias in Mental Arithmetic

In one of his seminal works, Stanislas Dehaene (2005) suggested that processing numerical magnitudes, such as in simple arithmetic, may employ “recycled” neural mechanisms of spatial attention operating on the mental representations of numbers, which is probably intrinsically spatially organized in the form of a “mental number line.” Close affinity between the mental representations of numbers and space is established in the current state of knowledge. One of the most prolific demonstrations of this affinity was Dehaene et al. (1993) study on so-called “SNARC” effect (spatial-numerical association of response codes), which indicates quicker reactions to relatively small numbers with the left hand and relatively large numbers with the right hand. Hence, the mental number line seems to be left-to-right oriented, at least in Western cultures, where left-to-right script is used (c.f. Göbel et al., 2011, for discussion of cultural and contextual variability of the mental number line), and numbers automatically activate corresponding spatial positions on this line. If so, the addition of natural numbers may require moving internal attention rightward (from smaller to larger numerical magnitudes), while subtraction may require leftward motion of attention. Indeed, it is worth noting that careful analyses by Cipora et al. (2018) and Toomarian and Hubbard (2018) also showed several other factors that may affect spatial-numerical associations.

Perhaps one of the strongest proofs for the involvement of spatial attention in number processing and arithmetic comes from the research on hemineglect – a syndrome of attentional ignoring of one side of the visual field (typically the left) after a contralateral lesion in the parietal cortex (for more detailed analysis of the symptoms and the neural bases of hemineglect, see Vallar and Bolognini, 2014). The research showed that patients with left visual field neglect ignore small numbers (below five). For example, when asked to state the number lying in the middle between one and nine, they may provide the answer seven, despite five being the correct value. Moreover, the patients have preserved addition skills but make more subtraction errors compared to healthy subjects or to patients with lesions in the right hemisphere who do not have neglect (Dormal et al., 2014). Right visual field neglect is relatively rare; however, case studies by Masson et al. (2017) demonstrated deficiencies in both large number processing and addition in such patients.

Increasing numbers of studies with healthy subjects also show contrasting patterns of leftward vs. rightward cuing of attention on addition vs. subtraction. For example, Mathieu et al. (2016) demonstrated that presenting a second operand on the left side of the screen sped up participants’ reactions in one-digit subtraction, while placing a second operand on the right side quickened reactions in addition.

Other research suggests that arithmetic operation may prime attention to the left or to the right. For example, in Masson and Pesenti (2016), study participants solved arithmetic problems and were then asked to detect a star presented either on the left or on the right side of the screen. Solving subtraction problems enhanced target detection on the left, while addition improved reactions to targets presented on the right side. Similar effects of directing attention through arithmetic operation (subtraction to the left and addition to the right) have also been documented by other researchers (Liu et al., 2017; Zhu et al., 2018, 2019). In another study with dual-task procedure (Masson and Pesenti, 2016) a distractor placed on the left impaired subtraction, while one placed on the right impaired addition. Taken together, these studies show evidence toward bidirectional interference between numerical operations and spatial attention.

### Operational Momentum as Numerical Magnitude-Related Bias and Its Relation to the Spatial-Numerical Biases

There is, however, another prominent bias in mental arithmetic. McCrink et al. (2007) demonstrated that when processing non-symbolic addition and subtraction (judging accuracy of the outcome of two summed or subtracted visual sets), people tend to accept overly large sets as outcomes of addition and overly small sets as outcomes of subtraction, which implies a tendency toward overestimating the results of addition and underestimation those of subtraction. The effect was later shown even in 9-month-old infants (McCrink and Wynn, 2009).

Knops et al. (2009a, b) integrated these two lines of research, demonstrating under-/overestimation biases in both symbolic and non-symbolic arithmetic, and additionally showed that both symbolic and non-symbolic arithmetic involve neural responses similar to those accompanying the saccadic eye-movement control processes. In particular, in the Knops et al. (2009a) study, a pattern classifier was trained in deciding between leftward and rightward saccades on the basis of the functional MRI scans of the parietal cortices of fifteen participants. Next, the classifier was presented with the scans of the brain activities of the same participants during arithmetic problem solving. The addition problems were classified as rightward saccades with above-random likelihood, while classification of the subtraction problems was at the random level (note that the participants in this study fixated their gaze centrally during arithmetic tasks, so no real saccades were involved). These results can suggest that the tendencies toward linking addition with the right-directed attention vs. linking subtraction with the left side and the tendencies toward overestimating the outcomes of addition vs. underestimating the outcomes of subtraction may be closely related and could involve the same mechanisms.

### Hypotheses Regarding the Mechanism of Operational Momentum

McCrink et al. (2007) proposed three explanations for the under-/overestimation bias in mental arithmetic. According to the first explanation, the bias is an effect of a simple heuristic, “addition is more, subtraction is less.” Such a heuristic may be interpreted either as a metaphorical description of some unknown mechanism or as a selection bias at the decision stage rather than the computation stage.

The second explanation assumes that overestimation in addition and underestimation in subtraction results from the properties of the spatial representation of numbers. The mental number-line is compressed (logarithmically scaled), while the arithmetic requires operating in uncompressed space (probably because it uses “recycled” spatial processes that evolved to process real space; cf. Dehaene, 2005). Inaccuracy of rescaling a compressed to an uncompressed representation (and vice versa) may, under some assumptions, cause typical under- or overestimation biases. In some way, this may be an instantiation of the mechanism underlying the heuristic “addition is more, subtraction is less.” However, as demonstrated in some later studies, such a heuristic does not work in some cases. The tendency toward under-/overestimating the results of subtraction/addition may sometimes be neutralized or even reversed (see e.g., Knops et al., 2013; Charras et al., 2014; Shaki et al., 2015; Blini et al., 2019). This makes both the heuristic and compression mechanism hypotheses problematic. Knops et al. (2014) constructed a computational model based on the imperfect uncompression hypothesis and compared its predictions to data collected from fourteen subjects performing numerosity estimation, numerosity comparison, and arithmetic (symbolic, non-symbolic, and cross-notational) tasks. At the group level, they found expected under-/overestimation biases; however, there was considerable individual variability, and individual precision of numerical magnitude representation (which is supposed to be dependent on scaling and compression/uncompression processes) did not correlate with the individual bias sizes in arithmetic. Thus, the biases may not be fully explained by the properties of numerical representation (i.e., scaling and compression).

The third alternative explanation assumes that the estimation biases are due to involvement of the spatial-attentional processes in mental arithmetic. Using the mental number-line metaphor, when subtracting or adding two numbers, one has to focus internal attention on the position of the first operand on the number line. Next, she/he moves attention by the distance proportional to the second operand’s magnitude to reach the unknown position of the result magnitude (McCrink et al., 2007). Visual attention has built-in a mechanism of anticipatory overestimation of the expected position of a moving object in the direction of motion. Freyd and Finke (1984) demonstrated such an anticipatory mechanism of attention. They found that people watching a sequence of still images of a moving object overestimate its future position in the direction of apparent motion when asked to select the next frame. In reference to momentum-based theory of motion in human “naive physics” this phenomenon was named “representational momentum” (“RM”). This mechanism, adapted for the purpose of mental arithmetic, may lead to underestimation in subtraction (setting focus too far in the direction of decreasing numbers), while the opposite may occur in addition. *Per analogiam*, McCrink et al. (2007) named the under-/overestimation bias in mental arithmetic “operational momentum” (“OM”).

### Representational Momentum as a Possible Mechanism of Operational Momentum-Related Bias

Momentum-like effects are common in attention and action. Hubbard (2015, 2017) analyzed different types of “momentum-like” effects in attentional processes and proposed that representational momentum is an important functional adaptation. When planning an action, e.g., reaching for an object, or planning a saccade directed at a moving object, we notice the object’s spatial position with a few tens of milliseconds of delay because the neural signal from the sensory input has to be transferred to the cortical level. Execution of the saccade or action would take another ten or even a few hundred milliseconds. Thus, at any given moment, the actual position of the object and the position represented in the brain are shifted relative to each other. To make the behavior efficient, the target’s position has to be anticipated. An overestimation of the target’s position proportional to the target’s speed may, therefore, provide optimal strategy in these cases, resulting in a representational momentum effect. Despite the fact that such a strategy may not be optimal in the case of arithmetic (usually, the precise outcome of arithmetic operation is desired), the same mechanism may produce the operational momentum effect (assuming the mechanisms of spatial attention are adapted or “recycled” for number processing). It should be noted, however, that this hypothesis also cannot easily explain the reversed operational momentum effect.

As Hubbard (2010) notes, there are a number of possible interpretations of the representational momentum mechanism, ranging from the low-level pereceptual-attentional mechanism to processes controlled by (implicit) conceptual knowledge. The explanatory status of representational momentum as an analog of operational momentum in arithmetic is then unclear. It may be either purely metaphorical, merely providing another description (like a model of a general attentional heuristic), or mechanistic, assuming use of the very same attentional processes in both spatial orienting and arithmetic (c.f. Hubbard, 2015, 2017). Under this more literal, mechanistic interpretation, RM and OM may share some computational and brain resources, which could lead to some interferences of attentional and numerical effects. Specifically, real motion of the attentionally tracked object may interfere with internal attention directed onto represented “motion” along the mental number-line and influence numerical processes, increasing or diminishing estimation biases in arithmetic. Some studies imply that this may indeed be the case. For example, perceiving leftward motion restores small numbers in space in left hemifield neglect (Salillas et al., 2009). It was also found that the direction of motion can cue attentional processes engaged in numerosity estimation. Schwiedrzik et al. (2016) used the neural adaptation paradigm in their experiment. Participants were adapted to leftward or rightward motion of a large cloud of dots. Adaptation to leftward motion led to overestimation of dot numerosity, while rightward motion led to underestimation biases. This suggests that the mechanism of attentional tracking leftward-moving objects sets attention to the small number side, while rightward motion moves attention to the larger number side (note that neural adaptation cancels direct effects of the stimuli, promoting the reversed pattern of reactions). The motion-induced operational momentum was also demonstrated in non-symbolic arithmetic in preschool children (Haman and Lipowska, 2021).

The interplay between representational and operational momentum mechanisms may, however, have further consequences. To effectively predict the future position of a tracked object, the representational momentum mechanism has to depend on the object’s velocity. Indeed, Freyd and Finke (1985), and Finke et al. (1986) documented velocity and acceleration effects on RM (see also Hubbard, 2015, 2017, for more extended review). However, as Hubbard states, the analog of velocity in operational momentum is unclear. Nevertheless, if the operational and representational momenta share a common mechanism, one may expect that not only motion itself but also velocity of motion may influence numerical processes and arithmetic. Thus, two possibilities may be considered. According to the first one, fast motion accompanying arithmetic operation may provide a cue for further shift of attention along the mental number-line in the direction of the operation, which should increase OM-related estimation biases. This hypothesis seems to be the most appealing one. We have noted above that directional attentional cuing (also involving moving stimuli) has a vivid effect on numerical processes and mental arithmetic. Typically, reaction times decrease in addition when attention is cued right and in subtraction when it is cued left. One may then expect that fast rightward motion should promote OM-based responses in addition, while fast leftward motion would enhance OM in subtraction. This expectation is based on well-established effects of priming and numerical distance. Comparing the size of two numbers at a greater distance on the number line is easier and requires less processing time than comparing two close numbers. In turn, priming the position on the mental number line facilitates access to a given number, as shown in both experimental studies and computer simulations (Zorzi et al., 2005).

There is, however, an alternative hypothesis that motion may be processed as a distractor competing for the same attentional resources. In this case, a fast rightward-moving stimulus would compete for attentional resources with numerical processes in addition, thus attenuating the internal attentional shift along the mental number-line and minimizing or even reversing the OM effect, while the same is expected for quick leftward motion in subtraction. At least one study (Masson and Pesenti, 2016) has demonstrated that presenting a distractor (albeit static) on the left side impairs subtraction, while a distractor located on the right impairs addition. Furthermore, the reversed effect of direction of motion on numerosity estimation, caused by neural adaptation induced by motion in the Schwiedrzik et al. (2016) study, may provide additional justification for this hypothesis. Nonetheless, this hypothesis seems to be less likely than the first one. Indeed, McCrink and Hubbard (2017) demonstrated that performing approximate arithmetic tasks under a loaded attention condition (in a divided attention task) even increased OM-related biases.

### Experimental Design Aimed at Testing the Hypothesis of a Common Attentional Mechanism of the Representational and Operational Momentum

The experiment described below is the first attempt to investigate the influence of speed and direction of motion on arithmetic. Thus, it was one of the first direct tests of the hypothesis of the common attentional mechanism of the representational and operational momenta.

We tested adult participants with two-digit addition and subtraction problems in which the operands moved across a computer screen either from the left to the right or vice versa. The participant’s task was to judge the accuracy of the proposed result. There were four levels of speed/velocity of the operand’s motion. According to the most prevalent hypothesis based on the common mechanisms for representational and operational momenta, we expected that increased velocity of moving numbers would also increase operational momentum. This can manifest either in decision times or increased acceptance of too-high outcomes in addition or too-low outcomes of subtraction, or in both. While we tested this hypothesis primarily, we also discuss alternative hypotheses in light of our results.

It should be clearly stated that such research design makes sense only if the mechanisms of both OM and RM are attentional. As mentioned above, interpretation of the momentum-like effects in terms of attentional processes is only one possible hypothesis in much wider range. In the case of a different nature of these mechanisms, our study does not lead to unambiguous predictions.

## Materials and Methods

### Participants

Twenty-four young adults (university students, 16 females, 8 males; 23 righthanded) with normal or corrected-to-normal vision voluntarily agreed to participate. They were informed only that the study concerns some aspects of numerical perception. All participants gave their written consent and received a small gift for participation. The procedure conformed to the requirements of the Declaration of Helsinki. Two additional participants did not complete the entire procedure and were replaced.

### Materials and Procedure

**FIGURE 1:**
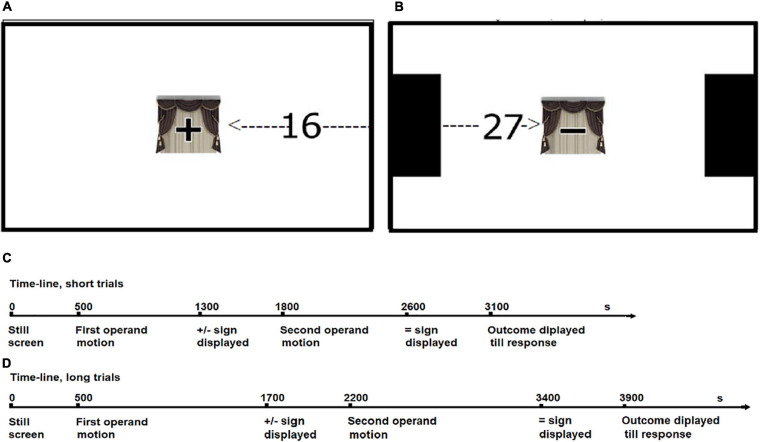
The trial layout and time-line. The dashed line with an arrow shows the path and direction of movement. **(A)** Screen layout, addition, long distance, and leftward motion. **(B)** Screen layout subtraction, short distance, and rightward motion. **(C)** Trial time-line of short-duration events. **(D)** Trial time-line of long-duration events.

Each participant watched 288 computer-generated animations, half of which presented addition problems, and the other half subtraction problems. The participant sat in front of the monitor so that the distance between the eyes and the screen was about 60 cm, but some uncontrolled variance in this regard was inevitable due to the differences in the posture of the participants and head movements during testing. A computer equipped with a 17” panoramic (9:16) LCD screen was used to run the experiment. The operands (two-digit numbers, 96 pixel font) entered the display either from the left or the right side (both operands in the same trial entered from the same side). They were then visible as they moved toward the center of the screen with varied velocity, until they were concealed behind a centrally located occluder (approximately 150 × 150 pixels) with an operator sign (plus or minus) displayed on it (see Figure 1). In half of the trials, the path of the moving operands was shortened by black rectangles displayed on both sides of the screen (the operands were starting from an edge of one of them). For each of the distances, motion lasted either 0.8 s (short events) or 1.2 s (long events). Combining event duration and distance resulted in four levels of operand motion speed: approximately 17 cm/s, 20.5 cm/s, 25.5 cm/s, and 30.5 cm/s (assuming that the mean distance between participant’s eyes and the screen was approximately 60 cm, angular speed levels may be approximated as 15.8, 18.3, 23.8, and 27.5 degrees per second, respectively). Five hundred milliseconds after the second operand was hidden, the occluder disappeared, revealing a number suggested as the outcome option. Such a time course of the event seems to be well fitted to the combined time course of mental arithmetic (Restle, 1970), RM (Freyd and Johnson, 1987), and mapping the number representation to space (Toomarian and Hubbard, 2018). Figure 1 illustrates the display and the course of the trials.

The subject then had to decide whether the outcome was right or wrong and pressed using dominant hand an appropriate key (“z” or “m”) on the computer keyboard, marked with colored stickers. For half of the subjects, the “correct” and “incorrect” buttons were switched. The selected response and reaction times (counted from the disappearance of the occluder to the moment of key-pressing) were recorded. There was a pause of 500 ms (still display) before the onset of the first operand, another pause between the first operand hiding completely behind the occluder and the appearance of the second operand, and a final pause between the hiding of the second operand and the unveiling of the outcome. The experimental procedure was programmed in C++ and was run under Microsoft Windows XP OS.

The stimuli consisted of 4 test blocks of 72 addition or subtraction problems (two addition and two subtraction blocks). We chose to block arithmetic operations, as they might partially activate different brain resources (Rosenberg-Lee et al., 2011); thus, mixing them together could lead to additional switching costs. The problems were randomly selected from a large pool of problems meeting the following conditions: (1) both operands and the outcome are positive two-digit numbers, and (2) only no-carry and no-borrowing problems were used. One-third of the problems in each block had a correct result (“c”) as the outcome, one-third had outcomes underestimated by 2 (“c−2”), and one-third of the problems had outcomes overestimated by 2 (“c + 2”). In the case of under- and overestimated outcomes, the outcome always belonged to the same ten as the correct result. We only used no-carry, no-borrowing problems because some work suggests the possibility that numerical units and tens are processed separately. In the case of carry or borrowing problems, predictions concerning spatial-numerical associations may be contradictory, depending on if they were based on ten or unit digit magnitude (Verguts and De Moor, 2005; Nuerk et al., 2015).

Each pair of problem and outcome was only used once in the subject’s individual set. The averages of larger operands in the subtraction problems and the outcomes of addition, as well as the mean distances between the second operand and the outcome in both operations, were equalized at the group level.

The blocks were arranged so that addition and subtraction blocks appeared interchangeably. Consecutive blocks were separated by a pause that allowed subjects to rest as long as they wished. In every block, there were 3 instances of every combination of the following varied factors: four speed levels (2 levels of event duration × 2 path lengths), the direction of movement (leftward or rightward), and magnitude of the proposed outcome (correct, too high, or too low). Hereafter we refer to these outcome options as “c” (correct), “c + 2” (too high) and “c−2” (too low), in respect to actual absolute numerical values of the outcome option. For example, in the case of both 57−22 subtraction problem and the 23+12 addition problem c = 35, c + 2 = 37, and c−2 = 33. Note that this notation is based on numerical values, not distance and direction of motion, and therefore may be somewhat confusing to readers more familiar with the literature on representational momentum. Eight different sets of four blocks were randomly constructed, each of them in three different orders, which resulted in 24 individual, fully counterbalanced sets. The first test block was preceded by an instruction screen and 8 warm-up problems with feedback. No feedback was given during the test trials. The entire session lasted approximately 25–30 min.

### Operationalization of Hypotheses

**FIGURE 2:**
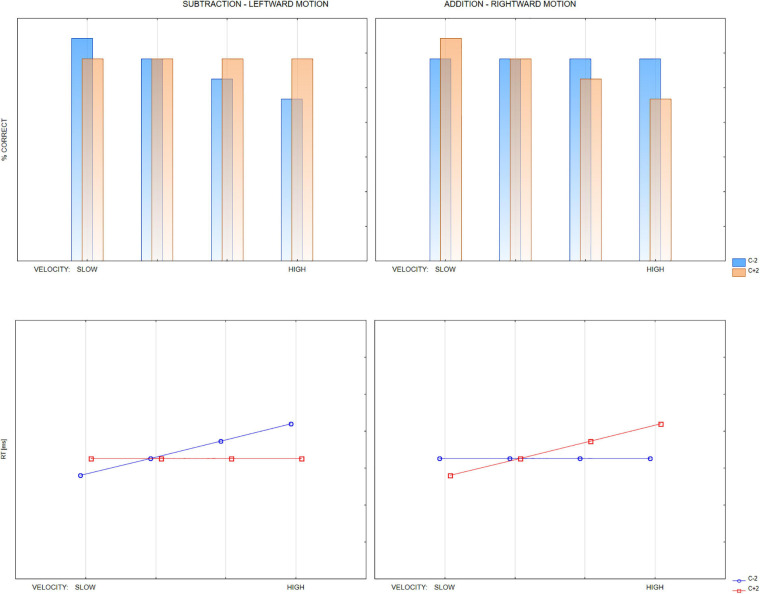
Hypothetical distributions of accuracy and reaction times, assuming a strict analogy hypothesis between OM and RM. 4-way interaction of the arithmetic OPERATION, DIRECTION OF MOVEMENT of the operands, VELOCITY of movement, and the OUTCOME OPTION (too low: c–2, too high: c + 2). Subtraction effects predicted only for leftward movement, addition effects only for rightward movement. Possible effects due to other factors (e.g., overall better performance in addition than subtraction) are not considered.

The design described above allows to test the hypotheses presented in the introduction. According to the most prevalent hypothesis based on the common mechanisms for representational and operational momenta, we expected that increased velocity of moving numbers would also increase operational momentum. Thus, we expected increased decision times of correct decisions or/and increased acceptance (decreased accuracy) in the case of too-high outcomes in addition and too-low outcomes in subtraction. It is important to note that we mean relative effects of comparison between too-high and too-low options (c + 2 and c−2) within the same operation (addition or subtraction). This is because each operation may add a separate effect. Particularly addition may be generally easier than subtraction, so processing addition problems may be generally quicker and more accurate. Figure 2 illustrates expected distributions of RTs and accuracy.

Furthermore, the direction of motion has to be considered, with rightward motion making it harder to reject too-high outcome options and leftward motion leading to the acceptance of too-low outcome options. Since previous studies indicated that rightward cuing of attention, or distraction in the right visual hemifield, influences addition, and the factors operating in the left part of the visual field influence subtraction, we may primarily expect the effects of rightward-directed motion on addition and leftward-directed motion on subtraction. Thus, our main hypothesis is that fast motion to the right would prime the position on the number-line ahead the correct one in the direction of motion (i.e., too-high number) and thus increase overestimation bias in addition, making it harder to judge the too-high outcome option as incorrect. This may be evidenced either in terms of increased RTs or decreased accuracy, or both. Quick motion to the left would increase underestimation bias in subtraction, making it harder to decide on too-low outcome options. More generalized interaction is, however, also possible, with both directions influencing both operations (in the opposite way).

Such operationalization is grounded in many previous studies on numerical representations showing the effects of numerical distance and priming of a position on the mental number line (for review, see Zorzi et al., 2005; c.f. also van Opstal and Verguts, 2011, for the empirical and computational investigation of the priming and distance effects in the “same-different” task, which is particularly relevant to our experimental design, as it matches the decisions required from our participants). As noted previously, comparing the size of two numbers at a greater distance on the number line is easier and requires less processing time than comparing two close numbers. In turn, priming the position on the mental number line facilitates access to a corresponding number. Assuming this, we expect that the shift of attention focus, associated with representational momentum, will cause a corresponding shift of internal attention along the mental number line and prime the expected outcome ahead of the correct one in movement direction. It should be noted that most of the studies to date have used selection of the outcome option rather than a decision on its correctness. In such a procedure it is not clear what the effects of movement along the mental number line should be, but it cannot be ruled out that it should be the opposite effect (and this was usually obtained, e.g., Knops et al., 2009a, b). Finally, because only no-carry operations were used, the subjects’ task was relatively easy: adding/subtracting a unit digit is enough to judge the correctness of the outcome, and the majority of participants realized this during the experiment. For this reason, we expect more salient effects in the analysis of response times rather than in accuracy.

According to the representational momentum-based hypothesis, the speed factor is expected to increase both OM effects: under/overestimation magnitude OM and direction-related OM. Thus, three separate interactions involving speed may be expected: (1) Speed × Operation × Outcome option and (2) Speed × Direction of motion × Outcome option, and (3) Speed × Direction of motion × Operation. However, because direction is known to interact with operation, more complex patterns (particularly an interaction of all four factors) are also possible. In these case, however, it should incorporate the effects listed above.

## Results

Responses given within less than 250 ms after outcome unveiling were deleted as “false-starts” (there only were three such RTs in the entire dataset: two responses to correct outcomes and one response to C−2), and RTs were then standardized, with reactions exceeding the individual mean by more than 3 SD (z > 3) eliminated from the analyses (3-14% of each participant’s responses, 7% on average, randomly distributed across different response categories). Standardization of the RTs allowed minimization of the effects of individual differences in the speed of processing on error variance. Only correct responses were included in the analyses of RTs; however, assuming high accuracy of responses (see below), the results for correct-only and all responses were convergent.

The analyses presented below were run on trials with under- and overestimated outcome options only (“c−2” and “c + 2”). There are at least two reasons for excluding correct (“c”) outcome trials from these analyses. First, the hypotheses concern too-low and too-high outcomes only. There is no clear expectation of how motion may affect processing correct outcome options. Second, separate response keys were assigned to the correct decisions in trials with incorrect vs. correct outcome options. Thus, these conditions may not be fully comparable (especially in the case of RT analyses). However, we also ran analyses on the full set of data, and the results did not diverge significantly from those presented below. Despite this, we also decided to include the distributions of the accuracy and RTs to the correct outcome trials in some figures to make this information available for the readers. Complete tables of means and standard errors (accuracy and reaction times) for all combinations of results are provided Supplementary Material.

### Analysis of Accuracy of Responses

The task was relatively simple, so the overall accuracy was high. Because only no-carry problems were used, most of the subjects realized during the session that the problems could be solved by simply adding or subtracting the unit digits and comparing the result to the last digit of the outcome option. We trimmed not only false starts (< 250 ms) but also too long reactions (> 3 SD above individual mean), assuming that they most likely result form distraction and may therefore not be reliable. After trimming the reactions with excessive times, the accuracy ranged from 0.844 to 0.976 (mean = 0.913, *SD* = 0.046). Indeed, all subjects made some errors (minimum seven) in at least seven or more categories of trials (design cells), and although there were no apparent outliers, the participants differed in the proportion and distribution of errors. We also checked the shape of error distribution and sphericity of variances and decided that they were sufficient for analysis of the error rate with GLM statistics (with Greenhouse-Geisser correction, which was required only in case of the main effect of speed). We used Statistica v. 13 software (Tibco Software Inc., United States).

Four-way repeated-measures ANOVA: 2 (Operation: subtraction vs. addition) × 2 (Direction of motion: leftward vs. rightward) × 4 (Speed of motion: from 1-slowest, to 4-quickest) × 2 [Outcome option: too low (c−2), too high (c + 2)] revealed significant main effects of two factors: (1) Operation, with responses to addition problems being more accurate [Madd = 0.939, SE = 0.010, Msub = 0.902, SE = 0.011; *F*(1,23) = 14.24, G-G epsilon = 1, *p* < 0.001, eta2p = 0.382], and (2) Speed [speed listed in increasing order: M1 = 0.943, SE = 0.008, M2 = 0.931, SE = 0.011, M3 = 0.910, SE = 0.013, M4 = 0.898, SE = 0.013; *F*(3,23) = 6.92, G-G epsilon = 0.697, corrected *p* < 0.001, eta2p = 0.255]. Note, however, that the two slower speeds differ from the two higher speeds by the duration of the movement, where the lower speed in each pair is associated with a shorter path (distance). Thus, the speed effect can be seemingly reduced to the effect of the event duration, as the Bonferroni-corrected *post hoc* tests revealed significant or near-significant differences between shorter- and longer-lasting motion trials (p1vs3 = 0.007, p1vs4 = 0.003, p2vs3 = 0.067, and p2vs4 = 0.033), but not between trials of the same duration (both ps = 1). As stated in T. Hubbard’s (2015, 2017) reviews, duration of the movement does not matter for strength of RM; thus, this effect is neutral for RM-based explanation of OM. On the other hand, a shorter trial duration means a greater time stress, which in turn leads to a more automatic allocation of attention and less deliberate decisions (Schneider and Shiffrin, 1977). It should be noted, however, that although the speed differed depending on the distance in short-duration trials, this difference does not affected the accuracy of decisions.

**FIGURE 3:**
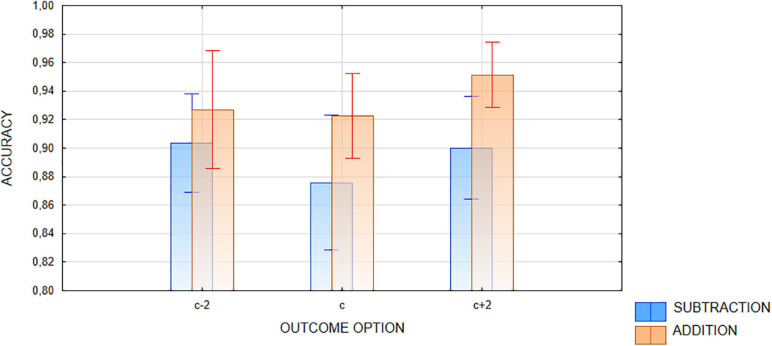
Response accuracy. Interaction between OPERATION (addition vs. subtraction) and OUTCOME OPTION (c + 2 vs. c–2) factors. Rods represent standard error. Correct outcome option was not included in the analysis and is added for illustration purposes only.

Only one interaction, Operation × Outcome option, appeared to be significant [*F*(1,23) = 5.24, G-G epsilon = 1, *p* < 0.035, eta2p = 0.179]. Bonferroni *post hoc* tests revealed that accuracy in addition is significantly higher than that in subtraction in the case of too-high outcome trials (*p* < 0.001). Even more importantly, for addition, the difference in accuracy between c−2 and c + 2 trials also approached significance (*p* = 0.065; Bonferroni corrected). Since this contrast was expected from our hypotheses and from previous OM studies, the planned comparison approach may be applied here, which in this case makes the contrast significant [*F*(1,23) = 4,385, *p* < 0.05]. No difference was found for c−2 and c + 2 outcome trials in subtraction [*F*(1,23) = 0.17, *p* = 0.74]. The results are visualized on Figure 3. This interaction seems to show some form of operational momentum, but it is not consistent with the representational momentum-based hypothesis. As stated previously, the RM-based hypothesis predicts that attentional movement along a mental number line overshoots the correct result, which should lead to hindering accuracy judgments of too-high outcome options in addition and too-low outcome options in subtraction. Participants in our experiment were better at recognizing too-high outcomes in addition as incorrect, while no difference was revealed in subtraction.

Although the required computation was easy, the task as a whole was relatively demanding on attentional resources, requiring from participants continuous tracking of the numbers moving on the screen, which, according to McCrink and Hubbard (2017), should also increase the bias, especially in the case of shorter duration trials. Unfortunately, direct comparison of our results to the previous studies is problematic, as most of them used a choice from a large set of outcome options rather than judging the correctness of a single proposed outcome. These two tasks pose different requirements on decision making processes. However, most importantly, none of the expected interactions involving the speed factor was found, which also does not support the RM-based explanation of the operational momentum effect.

### Analyses of the Reaction Times

Repeated-measures ANOVA 2 (Operation: subtraction vs. addition) × 2 (Direction of motion: leftward vs. rightward) × 4 ([Speed of motion (from 1 – the slowest, to 4 – the fastest)] × 2 (Outcome option: c−2 vs. c + 2) on response times revealed the main effects of Operation [*F*(1,23) = 31.71, *p* < 0.0001, eta2p = 0.580] and Outcome option [*F*(2,46) = 6.79, *p* < 0.016, eta2p = 0.228]. Addition was processed faster than subtraction, and too-low outcome options were processed quicker than too-high ones, which is generally consistent with previous findings in numerical cognition but not related to the hypotheses tested in our study. More importantly, there were also two significant interactions: Outcome option x Direction × Speed [*F*(3,69) = 3.36, *p* < 0.025, eta2p = 0.127], and the interaction of all four factors [*F*(3,69) = 2.89, *p* < 0.05, eta2p = 0.111].

**FIGURE 4:**
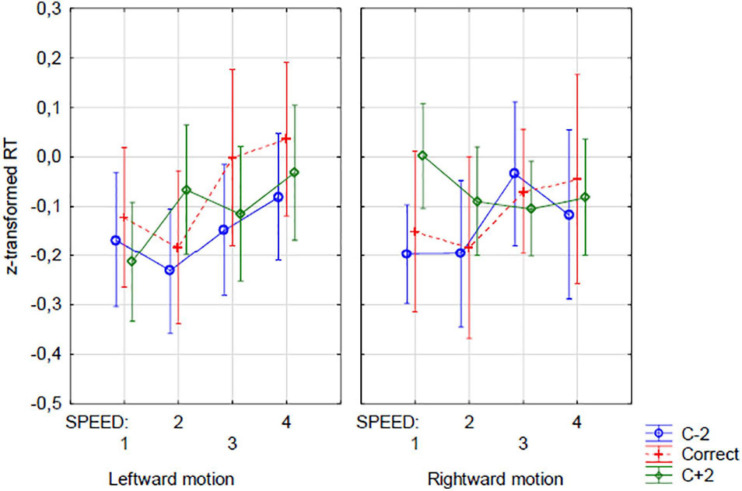
Reaction times. The interaction between DIRECTION OF MOVEMENT, OUTCOME OPTION, and VELOCITY (speed) factors. Blue line, c–2 option; Green line, c + 2 option; correct option (c) additionally plotted with red dashed line.

The presence of these two interactions seems to confirm findings from previous studies, showing the interference of motion-induced attention with numerical processes, and extends them onto velocity-related aspects of motion. The interpretation of these interactions is not, however, straightforward. Assuming common mechanisms for the RM and OM effects, our hypotheses predicted increased RT for c−2 option in the trials with left-directed movement of increased speed and in subtraction and for c + 2 option in the trials with rightward movement of increased speed and in addition. However, since the increased speed led to higher RTs itself (likely because of increased attentional load), the above effects should be considered relative rather than absolute. The results presented in Figure 4, illustrating the Direction of motion × Outcome option × Speed interaction, do not seem to confirm the hypothesis about the effects of direction of motion. In the case of the left-directed movement, all RTs generally increase with speed, and the only differences between too-high and too-low outcome options concern differences between slowest speed levels, which seems not to be related to RM-like mechanisms. For c−2 type outcomes, the RTs decrease between the two slowest speeds and then increase constantly. Transversely, RTs increase most between the first two (slowest) levels of speed for c + 2 type outcomes. Moreover, the pattern for rightward motion results seems to overtly contradict the hypothesis, with RTs decreasing for too-high outcomes and increasing for too-low outcomes in relation to increasing speed.

#### Analyses of the Effects Involving Outcome Option on RTs

**FIGURE 5:**
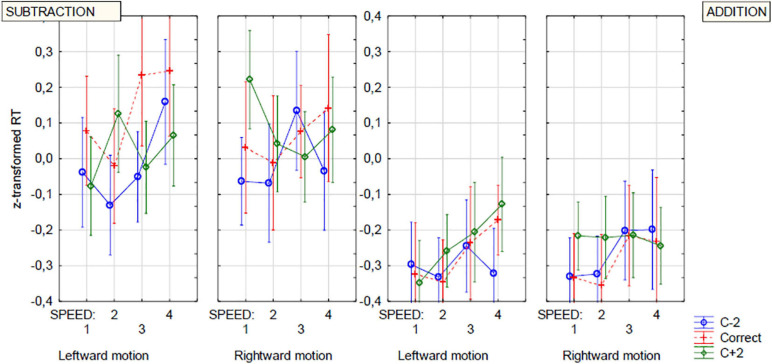
Reaction times. Four-way interaction of OPERATION, OUTCOME OPTION, VELOCITY (Speed), and MOVEMENT DIRECTION. Blue line, c–2 option; Green line, c + 2 option; correct option (c) additionally plotted with red dashed line.

Closer inspection of the four-way interaction illustrated in Figure 5 reveals different pictures for addition and subtraction, with the pattern of results for both operations being very complex. One way to disambiguate the pattern is to reduce the number of factors. The main manifestation of the OM effect (in respect to numerical magnitude) is the operation-dependent difference in responses (or response time) in the trials with too-low and too-high outcome options. By using this difference (instead of the response times themselves) as an operational momentum indicator, it is possible to reduce the number of factors in a complex analysis design and thus make the scheme a little simpler and clearer. We computed the differences between the mean standardized response time to the c−2 and c + 2 outcome types (MeanStRTc−2 - MeanStRTc + 2). The faster recognition of too-low outcomes as incorrect is shown by the negative values of the new dependent variable. Positive values indicate quicker reactions for too-high outcomes. Thus, according to the hypotheses based on the close analogy between OM and RM, we expected negative values of this indicator in rightward trials and in addition and positive values in leftward trials and in subtraction.

We ran a three-way repeated-measures ANOVA with Operation, Speed, and Direction of movement as the within-subject factors and this new variable as the dependent measure. Such an analysis allowed us to check how the operation, speed, and direction of motion factors could influence magnitude-related OM. Note that this analysis cannot fully substitute the one previously presented. However, it covers all significant effects found in the first analysis. As in the previous analysis neither Operation [*F*(1,23) = 0.111, *p* = 0.741, eta2p = 0.005] nor Direction of motion effect [*F*(1,23) = 0.167, *p* = 0.606, eta2p = 0.007] were significant in isolation from the Speed factor. For the Speed factor alone, the effect only approached significance [*F*(3,69) = 2.643, *p* = 0.056, eta2p = 0.103] and, alike the accuracy analysis, it seems to be the effect of trial duration rather than speed itself. The difference between response time to the trials with too-low and too-high outcomes was almost null for shorter-lasting trials but was negative in longer-lasting trials. Hence, the effect does not indicate any form of OM.

More importantly, there were two significant interactions, both involving the speed factor. There was the Direction of motion x Speed interaction [*F*(3,69) = 3.358, *p* < 0.025, eta2p = 0.127] and the interaction of all three factors [*F*(3,69) = 2.886, *p* < 0.05, eta2p = 0.111], which correspond to the interactions found in the previous analysis. However, the interaction between Operation and Speed, also expected from the RM-based hypothesis, was not significant [*F*(3,69) = 1.444, *p* = 0.238, eta2p = 0.059].

Closer examination of the results suggests that the Direction of motion x Speed interaction did not reveal any hypothesized OM-like effect. For both directions, the relationship with speed is similar in the three faster speed levels, with negligible differences. Only at the slowest speed did the difference become larger; however, none of the Bonferroni-corrected paired *post hoc* tests were significant.

The all-factor interaction seem to be more interesting. The pattern of this interaction is, however, complex, and a paired *post hoc* analysis did not reveal any significant contrasts between specific design cells. However, one should remember that the only way to manipulate speed is to manipulate movement duration or distance (or both). Unfortunately, both time and distance can be independent factors affecting mental arithmetic (but see Hubbard, 2015, 2017). Of these two variables, timing seems to be more important. More or less time pressures can result in more primitive, automatic, or less deliberative processes. Our hypotheses relate rather to automatic processes, so the results obtained in shorter trials seem to be more reliable. The possible effects of distance seem less obvious. While the mapping between line length and numerical quantities has been demonstrated in many tasks, such as line bisection or the number-line estimation task (e.g., Longo and Lourenco, 2007), it does not seem likely that the physical distance and distance on the number line are scaled in directly corresponding units. Moreover, in our task, the difference between the operands and the result varies to a great extent, thus requiring the rescaling of the mapping between the physical distance and numerical quantities in each sample. It can therefore be carefully assumed that since the complete picture is ambiguous, the most reliable indicator of the possible effects of movement speed on mental arithmetic will be to compare the short path and the long path in short-duration trials. In these trials longer distance corresponding to higher velocity.

**FIGURE 6:**
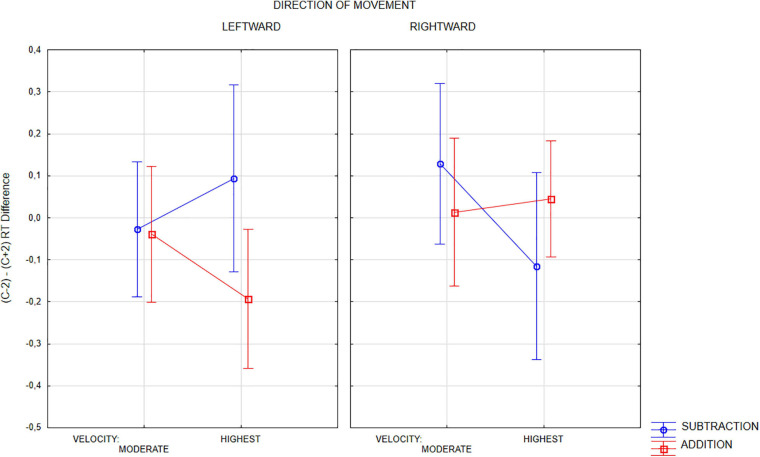
Three-way interaction of OPERATION, VELOCITY (speed), and MOVEMENT DIRECTION with the differential index of RT (c–2) - (c + 2). Only short-lasting trials included. Positive values of the differential index mean that in a given condition, the response to a too low outcome option (c–2) took a longer time than the response to an too high outcome option (c + 2).

We run three-way (2 Operation x 2 Movement direction x 2 Velocity (short/long distance)) repeated-measures ANOVA only on short duration, i.e., higher time pressure, trials. Only interaction of all three factors appeared to be significant [*F*(1,23) = 4.93, *p* < 0.037, eta2p = 0.176; for any other factor or interaction Fs < 1.9, *p* > 0.18]. This interaction could support the hypothesis of the effects of the velocity of physical motion on mental arithmetic as a function of operation and direction of motion, however, as Figure 6 shows, only in the case of subtraction this effect is consistent with the predictions of the hypothesis about the joint mechanism of RM and OM. In subtraction, participants tended to faster rate too high (c + 2) rather than too low (c−2) outcomes fast motion, when the motion was directed to the left, and the opposite tendency was observed in the case of fast right-directed movement. It was shown by positive values of the *MeanStRTc*−*2 – MeanStRTc* + *2* index in the case of leftward movement, and negative in the case of rightward movement. However, in the case of addition, there has been a trend toward shorter c−2 outcom evaluation times with fast left-directed movement and no clear effect with fast right-directed movement, which is not in line with the main hypothesis. In general, however, the interpretation of these results should be approached with caution, because in the *post hoc* test with Bonferroni correction, no contrast was significant (the lowest *p* > 0.45). Although, as noted earlier, short-duration trials seem to be more reliable for the hypotheses made, we tested also long-duration trials. In this case, however, neither the effect of the arithmetic operation nor any interaction containing this factor approached statistical significance.

## General Discussion

### Summary of the Results

In summary, we have found an operational momentum-like effect in the analysis of response accuracy (at least in addition). Participants were more efficient in recognizing too-high than too-low outcomes of addition as incorrect. This effect, although consistent with the previous studies, does not fit the representational momentum-based hypothesis. Moreover, in this analysis speed did not interact with the numerical factors, although the main effect of speed alone was significant (which seems to be related to general attentional load caused by quick motion).

Analyses of reaction times revealed a more complex pattern of results. We obtained a number of statistically significant effects, including those involving the speed factor, which shows that our design was suitable for testing the hypotheses concerning the relation between external spatial attention and mental arithmetic. However, while each of these effects can be meaningfully interpreted individually, together they do not provide a coherent picture and only some of them confirm the hypothesis about the common mechanism of RM and OM, while others contradict it. In the case of addition, quick rightward motion quickened evaluation of c + 2 outcomes, while quick leftward motion enhanced processing c + 2 outcomes in subtraction. Only this second effect conforms the RM-based hypotheses. Thus, alternative hypotheses should also be considered.

### Analysis of Accuracy

After analyzing the results of accuracy judgments, it seems that none of the motion-related factors (speed and direction) interacted with number-related factors (operation and outcome option). There was the main effect of speed, which can be reduced to the duration of the event. Accuracy was improved in longer-lasting trials, which seems trivial, as subjects had more time to conduct the calculation (an analogous effect of speed has been demonstrated in some response time analyses). Importantly, as shown in Hubbard (2015, 2017) review, distance and duration are not factors that significantly influence representational momentum and therefore cannot be part of the common RM and OM mechanism. The only significant interaction did not include any motion-related factor and showed that too-high addition results are recognized better than too-low ones, with a weak, non-significant reverse trend in subtraction, results that were contrary to what was predicted, i.e., that representational momentum would move the expected result further on the number-line in the direction of operation and thus make it harder to recognize too-high outcomes in addition and too-low outcomes in subtraction.

Our results are more consistent with another version of the attentional explanation of OM. Operation may prime attention to the operation-consistent direction on the mental number line (larger numerical magnitudes in addition and smaller magnitudes in subtraction) and thus enhance operating in this part of the numerical space, at least making decisions about higher outcomes easier in addition. Subtraction outcomes are located on the “small number” or “left” part of the number-line, which primes attention by default because of either cultural (e.g., left-to-right script direction; cf. Azhar et al., 2020; Masson et al., 2020), or biological factors e.g., right hemisphere dominance in spatial attention, which results in left visual hemifield prevalence and has been well documented in the literature on attention (Corbetta and Shulman, 2002; see also Toomarian and Hubbard, 2018). If so, no strong cuing effect is to be expected for subtraction. Note also, that in most previous studies (e.g., Knops et al., 2009b), participants were asked to choose the result from a set of options. In this case, both the hypothesis proposed above and the hypothesis based on representational momentum lead to similar predictions. True/false decisions based on actually displayed numbers and expected outcomes were forced in our study, which consequently may explain the divergence of our results with previous works.

### Response Time Analysis

At least some results of RT analyses may also support the hypotheses diverging from that appealing to the representational momentum mechanism. The main effect of the outcome option factor revealed faster responses to lower (c−2) outcomes. This may be a manifestation of the size effect reported in research on number processing (c.f. Feigenson et al., 2004; Dehaene, 2011), or it may be the result of a default setting of the internal focus of attention on small numbers (on the left side of the number line), which does not contradict the previous explanation. However, the analyses of reaction times also revealed a more complicated picture. In addition to findings from previous studies, which showed relationships between RTs in arithmetic and spatial directions (left/right), outcome size, and operation, our study also revealed that these relationships are modified or even radically altered by the speed of motion. Indeed, although the prediction of interactions involving a speed factor was the most innovative part of our test of the common RM and OM mechanism hypothesis, the whole pattern of these interactions does not seem to be consistent with predictions derived from this hypothesis. Only the effect of the interaction of speed and direction of movement on the processing time of too-high and too-low outcome options in subtraction (enhanced processing of too-high outcomes in the fastest trials with leftward motion) seems to conform the RM-based hypothesis. Some early works on OM (e.g., McCrink et al., 2007) also showed this effect only for subtraction. However, many later works revealeded a stronger effect for addition (e.g., Masson et al., 2018). The inverted pattern of results in addition contradicts the common OM and RM mechanism hypothesis. So, assuming that the common mechanism hypothesis is insufficient, we discuss other alternative explanations.

### Alternatives to the Main Hypothesis

#### Addition and Subtraction Involve Spatial Attention Differently

Reverse patterns of the speed effect on the RTs in Direction x Outcome option and Direction x Outcome option × Velocity interaction in addition and subtraction may be caused by differences in processing of symbolic addition and subtraction in the human brain. Some studies indicate that addition relies on memory and symbolic representation; thus, they activate the left inferior parietal lobe (including the supramarginal gyrus and angular gyrus), which is linked to linguistic fact retrieval and symbolic number processing. Therefore, addition may be less dependent on the original representations of the number and their relationship to space. Subtraction, on the other hand, requires more magnitude processing, and thus may rely on common spatial and numerical brain areas (Rosenberg-Lee et al., 2011; but see Arsalidou and Taylor, 2011, for a meta-analysis showing a more complex picture). Under this hypothesis, the representational momentum-based mechanism of operational momentum may be restricted to, or at least be more pronounced, in subtraction. In line with this suggestion, some early works reported a stronger OM effect in subtraction than addition (e.g., McCrink et al., 2007). However, several criticisms of this interpretation may be raised. First, even in the case of subtraction, the pattern of results does not clearly support an RM-based hypothesis. There was no OM-like effect in the analysis of response accuracy in subtraction, whereas it was quite robust in addition. In the analysis of RTs in addition trials, a clear pattern of speed effect was revealed, which contradicts the RM-based hypothesis. Second, contrary to McCrink et al. (2007) and other early studies indicating a stronger OM in subtraction than addition, most experiments that tested OM in the context of directional attentional biases (either leftward or rightward oriented) reported effects for both operations. Moreover, rightward bias in addition was typically more robust, and in a few cases, no leftward bias in subtraction was observed (Masson et al., 2018). In the most characteristic study by Knops et al. (2009a), brain activity during addition was classified as a rightward saccade, while no leftward bias for subtraction was found. Note, that the classifier was presented with activity within the bilateral superior parietal lobes, which, in previously mentioned studies on brain correlates of arithmetic, were found to be more active in subtraction. Thus, we can conclude that stronger involvement of memory retrieval and symbolic processes in addition does not switch off spatial-numerical processes related to operational momentum.

#### Motion Was a Distractor Rather Than a Cue for Number-Oriented Attention

Our predictions assumed that attentional shift induced by motion would add to attentional movement along the mental number-line. Thus, fast motion in an operation-consistent direction (leftward in subtraction, rightward in addition) would set the focus of attention too far on the mental number-line, increasing OM bias. This prediction was based on previous studies of the involvement of spatial attention in arithmetic (Dormal et al., 2014; Mathieu et al., 2016; Masson et al., 2017), as well as studies on the distance effect and priming in numerical representations Zorzi et al., 2005; van Opstal and Verguts, 2011). It is possible, however, that real movement of numerals along the screen, and the representational momentum produced in this way, may deliver a distractor rather than a cue for numerical processes (cf. Masson and Pesenti, 2016). It is not clear what effect may be expected in this case. The most straightforward prediction may be that fast motion would generally impair arithmetic processing. The main effects of speed in both accuracy and RTs analysis, which in large part can be reduced to a trial duration effect, support this explanation. Reaction times became longer, and the correctness of the responses decreased as the speed of movement increased, and particularly with decreased duration. However, these effects in isolation from the Operation, Direction, or Outcome option factors only demonstrate partial involvement of the same general attentional resources in both arithmetic and motion processing, but not representational momentum effect on mental arithmetic. Thus, they are neutral regarding the objectives of our study.

Another version of the motion-as-distractor hypothesis is also possible. The involvement of attention attracted by an object or movement in a given part of space may hinder access to this region by other processes (e.g., attention directed to numbers), as shown by Masson and Pesenti (2016), or in the neural adaptation experiment (Schwiedrzik et al., 2016). This should lead to reversing a pattern of results predicted by the positive version of the RM-based hypothesis. In our study, this pattern of results was found in the analysis of the effect of the fastest speed levels in the case of addition, but not subtraction.

Either way, none of these versions of the distraction hypothesis is able to accommodate the cases of enhanced RTs found in the trials with increased speed. While the RTs generally grew with increasing speed, in some conditions and cases, the reverse pattern was also observed (particularly for too-high outcomes in the rightward-motion condition, but see Figures 4, 5 for some other cases).

#### Misdesigned Manipulation of the Movement of Operands

One possibility that should also be considered is that a form of movement such as in our design does not induce representational momentum. Most RM studies used circular motion, and it was apparent (composed of images of the object in shifted positions), not smooth and linear motion. It has sometimes been argued that this is a condition for obtaining RM (Kerzel, 2003). However, Hubbard (2015) discusses this thesis and gives examples of studies in which smooth linear motion induced RM. Additionally, taking into account the linear nature of the number representation, the hypothesis of the analogy between RM and BO2 rather requires a linear movement.

#### Misdesigned Manipulation of the Direction of Movement

We should also consider the possibility that our operationalization of the direction of motion factor was incorrectly planned. Rightward movement began on the left side of the screen and ended in the center of the screen (leftward motion was symmetrical). Assuming the participants were fixating their eyes in the center of the screen, a movement to the right could activate the left visual field, and a movement to the left could activate the right visual field. It seems unlikely. Indeed, participants did not know which side the number would appear on, so its appearance was likely to draw attention contralateral to the direction of motion. However, since the subjects were not instructed to fixate on the center of the screen and had to focus their eyes on a number to recognize it, tracking the moving number consistently should switch their attention in the direction of movement. Nevertheless, even if the manipulation of the direction of movement was not efficient, the results are still not in line with the assumption of the common RM and OM mechanism.

Another element of our procedure that may also be questioned is the relatively long (500 ms) interval between the second operand being covered and the result being revealed. RM reaches its peak effect after approximately 300 ms and then decreases and disappears after a much longer delay (above 700 ms; Restle, 1970). It seems, however, that 500 ms is an appropriate time here, taking into account that the numerical effects of spatial variables reveal themselves with an additional delay (projecting “numerical space” onto visual space needs some additional time (Ranzini et al., 2009). Conversely, a long interval may cause too large an overestimation of the spatial position and, on the basis of the inhibition of return mechanism, or “attentional momentum” (Pratt et al., 1999; Hubbard, 2015, 2017), make it difficult to access the overestimated outcome option on the number line. In such a situation, however, the results of the study should be consistent with the main hypothesis based on the analogy between OM and RM. Moreover, the timing argument, if relevant, should mostly apply to longer-lasting trials, whereas, as our analyses show, more pronounced effects were found in trials of shorter duration.

#### Intermediate Summary

To summarize, several significant interactions between numerically relevant factors (Operation and Outcome option) and spatially relevant factors (Direction and Speed of motion) were found in our study. This confirms the general phenomenon of operational momentum and emphasizes the role of recycled spatial attention in arithmetic processes. An important contribution of our research is not only confirmation of this relationship but also an indication that the properties of motion in space (speed and direction) partly shape this relationship and to some extent can be considered as real counterparts of imagined movement along the mental number-line.

The revealed pattern of these interactions does not, however, uniformly fit the hypothesis of common mechanisms of the representational and operational momentum effects, nor any known alternative explanation of OM. Previous studies used relatively simple experimental designs that allowed internally consistent results to be obtained. Inconsistencies only occurred between the results of different projects (e.g., standard vs. inverted OM and differences in OM in addition and subtraction). The simultaneous incorporation of various numerical (result option, type of arithmetic operation) and spatial factors (direction and speed of movement) into our design resulted in various combinations of factors. It was revealed that depending on the combination of these factors, interactions of attention and mental arithmetic could lead to opposite effects. Therefore, we have to consider the following question: what is the nature of the interaction of mental arithmetic and spatial attention revealed in our results? Unfortunately, any answer to this question would inevitably be speculative. Nevertheless, we make an attempt to find such an answer below.

#### Dynamic Mapping of Numbers Onto Space

Our final supposition is that although the link between spatial attention and numerical representation is substantial for arithmetic processes, which manifested itself in several significant effects in our study, it is not as direct as that predicted by the hypotheses of the mental number-line as the permanent representation of number in the brain. Although conceptualization of number representation as a mental number line is overly or implicitly accepted in many studies, some explicit computational models point to a more complex processing mechanism. In particular, in two studies aimed at computational modeling different aspects of numerical cognition, including the operational momentum effect (Chen and Verguts, 2012; Prather, 2012), permanent numerical representations are not inherently spatial. Instead, on-line number processing, arithmetic in particular, still is claimed to take place in the represented space using recycled attentional mechanisms. The representations of numerical magnitudes have both scalar and ordinal properties, which allow projecting numbers onto space. Constructing spatial representation of a numerical problem is, however, an on-line process, which may depend on several numerical and contextual factors (e.g., numerical problem size and distances between operands, external spatial cues and distractors, and linguistically and culturally constrained associations with space). Only in this *ad hoc* represented space can the spatial properties of number and spatial attention interact (in either bidirectional priming or negative interference process). Additionally, as discussed by van Dijck and Fias (2011), the process of mapping numbers onto space engages working memory resources; thus, any other information competing for these resources may influence the final spatial model of the arithmetic problem. The heterogeneous nature of number-space mapping, involving some inherent properties of number representation as well as both spatio-visual and verbal working memory constructions, was also indicated in the experiments by Koten et al. (2011). While specifically concerning OM, the model by Knops et al. (2014) also demonstrated that a single mechanism, especially based on spatial properties of basic numerical representations, may not be enough to fully explain this phenomenon. The dynamic construction of the internal spatial representation also manifests itself in other areas of core cognition. Fini et al. (2017) demonstrated that priming a biological motion that is congruent with the anticipated “goal” of the observed agent, changes extrapersonal space categorization, enlarging the portion of space judged as “near.” It should be noted that such an effect can also be categorized as “momentum.” A similar mechanism in arithmetic may explain the interaction of operation and direction of movement (left part of space as “near” in subtraction, right part of space in addition).

Such an approach, assuming dinamic mappings between space and numbers helps to explain why the interaction of spatial and numerical factors revealed in our study is not only significant but also highly heterogeneous. Regrettably, this approach is not constrained enough to allow us to explain the specific pattern of results revealed in our research. It may, however, be the starting point for much more detailed hypotheses that could be tested in appropriate experimental designs.

## Conclusion

The pattern of results as a whole seems to conflict (at least in large part) with the tested model, in which operational momentum is the exact counterpart of representational momentum or even utilizes the same specific attentional mechanisms. Although we have shown that direction and speed of motion are powerful factors modifying internal attention directed toward numbers, it does not appear that the perception of real movement directly impacts (“accelerates” or “slows down”) the imaginary movement of attention along the mental number line. It seems more likely that the visible movement of numbers can be a cue or a distractor for the processes of internal attention operating on the online constructed number space. Although some attentional mechanisms are “recycled” for use in numerical processes and, in particular, in arithmetic, and therefore, there is interference between spatial attention and numerical processes, this is not a direct mapping. Conversely, the spatial characteristics of the representation of numbers may not be its permanent property at all, but rather a flexible “on-line” construction build for the needs of computational processes using the aforementioned “recycled” spatial attention mechanisms.

Thus, although our study adds new evidence of the operational momentum, it does not provide fully conclusive results. It may, however, open a new window toward a future study of the process of on-line building of spatial representations of numerical problems and modeling the involvement of spatial attention mechanisms in solving these problems. Perhaps more advanced control over the numerical and spatial factors used in our study would allow determinations of the properties of these processes at their different stages.

## Data Availability Statement

The raw data supporting the conclusions of this article will be made available by the authors, without undue reservation.

## Ethics Statement

The studies involving human participants were reviewed and approved by Komisja ds. Etyki Badań Naukowych, Faculty of Psychology, University of Warsaw, Warsaw, Poland. The patients/participants provided their written informed consent to participate in this study.

## Author Contributions

MH designed general procedure, coordinated research and data processing, conducted statistical analyzes, and wrote the manuscript. HM prepared experimental materials, recruited participants, conducted research, and made initial data processing. MG scripted and implemented the procedure. All authors contributed to the article and approved the submitted version.

## Conflict of Interest

The authors declare that the research was conducted in the absence of any commercial or financial relationships that could be construed as a potential conflict of interest.
