# Supplementary material for: Perceived Motion and Operational Momentum: How Speed, Distance, and Time Influence Two-Digit Arithmetic
Source: Front Psychol. 2021 Jul 13;12:653423. doi: 10.3389/fpsyg.2021.653423 (PMC8313890; doi:10.3389/fpsyg.2021.653423)
Supplement: Supplementary file 1 [file Table_1.docx]

SUPPLEMENTARY MATERIALS PART A

Distribution of accuracy across all combinations of factors

| OPERATION | DIRECTION  L-left, R-right | VELOCITY  1-slowest,  4-quickest | OUTCOME OPTION | MEAN ACCURACY | SE |
| --- | --- | --- | --- | --- | --- |
| SUBTRACTION | L | 1 | c-2 | 0,965278 | 0,017316 |
| SUBTRACTION | L | 1 | c | 0,923611 | 0,022386 |
| SUBTRACTION | L | 1 | c+2 | 0,930556 | 0,022245 |
| SUBTRACTION | L | 2 | c-2 | 0,930556 | 0,026384 |
| SUBTRACTION | L | 2 | c | 0,881944 | 0,032476 |
| SUBTRACTION | L | 2 | c+2 | 0,944444 | 0,019210 |
| SUBTRACTION | L | 3 | c-2 | 0,826389 | 0,029213 |
| SUBTRACTION | L | 3 | c | 0,868056 | 0,036142 |
| SUBTRACTION | L | 3 | c+2 | 0,895833 | 0,031426 |
| SUBTRACTION | L | 4 | c-2 | 0,902778 | 0,026384 |
| SUBTRACTION | L | 4 | c | 0,909722 | 0,020012 |
| SUBTRACTION | L | 4 | c+2 | 0,847222 | 0,029957 |
| SUBTRACTION | R | 1 | c-2 | 0,937500 | 0,019588 |
| SUBTRACTION | R | 1 | c | 0,861111 | 0,027778 |
| SUBTRACTION | R | 1 | c+2 | 0,895833 | 0,029781 |
| SUBTRACTION | R | 2 | c-2 | 0,868056 | 0,034722 |
| SUBTRACTION | R | 2 | c | 0,861111 | 0,029534 |
| SUBTRACTION | R | 2 | c+2 | 0,909722 | 0,022386 |
| SUBTRACTION | R | 3 | c-2 | 0,923611 | 0,020012 |
| SUBTRACTION | R | 3 | c | 0,826389 | 0,041943 |
| SUBTRACTION | R | 3 | c+2 | 0,895833 | 0,029781 |
| SUBTRACTION | R | 4 | c-2 | 0,875000 | 0,027012 |
| SUBTRACTION | R | 4 | c | 0,875000 | 0,025080 |
| SUBTRACTION | R | 4 | c+2 | 0,881944 | 0,023483 |
| ADDITION | L | 1 | c-2 | 0,944444 | 0,019210 |
| ADDITION | L | 1 | c | 0,944444 | 0,016382 |
| ADDITION | L | 1 | c+2 | 0,958333 | 0,020682 |
| ADDITION | L | 2 | c-2 | 0,944444 | 0,019210 |
| ADDITION | L | 2 | c | 0,951389 | 0,015796 |
| ADDITION | L | 2 | c+2 | 0,965278 | 0,014114 |
| ADDITION | L | 3 | c-2 | 0,909722 | 0,024531 |
| ADDITION | L | 3 | c | 0,916667 | 0,024574 |
| ADDITION | L | 3 | c+2 | 0,965278 | 0,014114 |
| ADDITION | L | 4 | c-2 | 0,902778 | 0,028227 |
| ADDITION | L | 4 | c | 0,923611 | 0,026503 |
| ADDITION | L | 4 | c+2 | 0,965278 | 0,014114 |
| ADDITION | R | 1 | c-2 | 0,937500 | 0,024187 |
| ADDITION | R | 1 | c | 0,895833 | 0,024187 |
| ADDITION | R | 1 | c+2 | 0,972222 | 0,012951 |
| ADDITION | R | 2 | c-2 | 0,937500 | 0,022008 |
| ADDITION | R | 2 | c | 0,937500 | 0,019588 |
| ADDITION | R | 2 | c+2 | 0,951389 | 0,018713 |
| ADDITION | R | 3 | c-2 | 0,944444 | 0,019210 |
| ADDITION | R | 3 | c | 0,923611 | 0,022386 |
| ADDITION | R | 3 | c+2 | 0,916667 | 0,024574 |
| ADDITION | R | 4 | c-2 | 0,895833 | 0,029781 |
| ADDITION | R | 4 | c | 0,888889 | 0,031191 |
| ADDITION | R | 4 | c+2 | 0,916667 | 0,026543 |

SUPPLEMENTARY MATERIALS PART B

Distribution of z-transformed RTs across all combinations of factors

| OPERATION | DIRECTION  L-left, R-right | VELOCITY  1-slowest,  4-quickest | OUTCOME OPTION | Mean z-transformed RT | SE |
| --- | --- | --- | --- | --- | --- |
| SUBTRACTION | L | 1 | c-2 | -0,038142 | 0,074405 |
| SUBTRACTION | L | 1 | c | 0,077975 | 0,073797 |
| SUBTRACTION | L | 1 | c+2 | -0,077332 | 0,066762 |
| SUBTRACTION | L | 2 | c-2 | -0,130135 | 0,067618 |
| SUBTRACTION | L | 2 | c | -0,020826 | 0,077680 |
| SUBTRACTION | L | 2 | c+2 | 0,125862 | 0,079126 |
| SUBTRACTION | L | 3 | c-2 | -0,051163 | 0,061160 |
| SUBTRACTION | L | 3 | c | 0,234702 | 0,096227 |
| SUBTRACTION | L | 3 | c+2 | -0,024402 | 0,062522 |
| SUBTRACTION | L | 4 | c-2 | 0,159116 | 0,084434 |
| SUBTRACTION | L | 4 | c | 0,245602 | 0,088255 |
| SUBTRACTION | L | 4 | c+2 | 0,065173 | 0,068536 |
| SUBTRACTION | R | 1 | c-2 | -0,063461 | 0,059299 |
| SUBTRACTION | R | 1 | c | 0,031345 | 0,089180 |
| SUBTRACTION | R | 1 | c+2 | 0,221409 | 0,066489 |
| SUBTRACTION | R | 2 | c-2 | -0,068788 | 0,079974 |
| SUBTRACTION | R | 2 | c | -0,011913 | 0,091061 |
| SUBTRACTION | R | 2 | c+2 | 0,041483 | 0,064816 |
| SUBTRACTION | R | 3 | c-2 | 0,134127 | 0,080581 |
| SUBTRACTION | R | 3 | c | 0,075946 | 0,062553 |
| SUBTRACTION | R | 3 | c+2 | 0,005375 | 0,061195 |
| SUBTRACTION | R | 4 | c-2 | -0,034421 | 0,080533 |
| SUBTRACTION | R | 4 | c | 0,141679 | 0,099486 |
| SUBTRACTION | R | 4 | c+2 | 0,080772 | 0,071070 |
| ADDITION | L | 1 | c-2 | -0,297034 | 0,057557 |
| ADDITION | L | 1 | c | -0,323057 | 0,069177 |
| ADDITION | L | 1 | c+2 | -0,347492 | 0,056871 |
| ADDITION | L | 2 | c-2 | -0,331942 | 0,053234 |
| ADDITION | L | 2 | c | -0,345548 | 0,057031 |
| ADDITION | L | 2 | c+2 | -0,258452 | 0,049143 |
| ADDITION | L | 3 | c-2 | -0,244561 | 0,062491 |
| ADDITION | L | 3 | c | -0,236226 | 0,076237 |
| ADDITION | L | 3 | c+2 | -0,205857 | 0,067270 |
| ADDITION | L | 4 | c-2 | -0,320686 | 0,060638 |
| ADDITION | L | 4 | c | -0,172515 | 0,047295 |
| ADDITION | L | 4 | c+2 | -0,127896 | 0,063812 |
| ADDITION | R | 1 | c-2 | -0,330259 | 0,052442 |
| ADDITION | R | 1 | c | -0,333506 | 0,059838 |
| ADDITION | R | 1 | c+2 | -0,216501 | 0,046084 |
| ADDITION | R | 2 | c-2 | -0,323144 | 0,051039 |
| ADDITION | R | 2 | c | -0,355154 | 0,068986 |
| ADDITION | R | 2 | c+2 | -0,220972 | 0,055769 |
| ADDITION | R | 3 | c-2 | -0,201568 | 0,066811 |
| ADDITION | R | 3 | c | -0,215744 | 0,068206 |
| ADDITION | R | 3 | c+2 | -0,214567 | 0,057575 |
| ADDITION | R | 4 | c-2 | -0,198905 | 0,080982 |
| ADDITION | R | 4 | c | -0,231891 | 0,086622 |
| ADDITION | R | 4 | c+2 | -0,244249 | 0,051939 |
